# Survie et facteurs pronostiques du cancer primitif du foie à Cotonou (République du Bénin) de 2014 à 2020

**DOI:** 10.48327/mtsi.v4i3.2024.418

**Published:** 2024-07-18

**Authors:** Houéhanou Rodrigue GNANGNON Freddy, Aboudou Raïmi KPOSSOU, Koffi N'TCHA, Salmane Ariyah AMIDOU, Vincent ZOSSOU, Cosme TOUME, Rodrigue S. ALLODJI, Dismand Stephan HOUINATO, Dansou Gaspard GBESSI, Jean SEHONOU

**Affiliations:** 1 Clinique universitaire de chirurgie viscérale, Centre national hospitalier universitaire-Hubert Koutoukou Maga (CNHUHKM), Cotonou, Bénin; 2 Registre des cancers de Cotonou, ministère de la Santé, Bénin; 3 Laboratoire d’épidémiologie des maladies chroniques et neurologiques, Faculté des sciences de la santé, Cotonou, Bénin; 4 Institut national de la santé et de la recherche médicale ((INSERM) U1094, Institut de recherche et développement (IRD) U270, Univ. Limoges, Centre hospitalier universitaire de Limoges, EpiMaCT - Épidémiologie des maladies chroniques en zone tropicale, Institut d’épidémiologie et de neurologie tropicale, OmegaHealth, Limoges, France; 5 Clinique universitaire d'hépato-gastroentérologie, Centre national hospitalier universitaire-Hubert Koutoukou Maga (CNHU-HKM), Cotonou, Bénin; 6 Institut de formation et de recherche en informatique, (IFRIUAC), Cotonou, Bénin; 7 École polytechnique d'Abomey-Calavi, (EPAC-UAC), 01 P.O. Box 2009, Cotonou, Bénin; 8 Université Paris-Saclay, UVSQ, Univ. Paris-Sud, Inserm, Équipe radiation epidemiology, CESP, 94805, Villejuif, France; 9 Institut national de la santé et de la recherche médicale (INSERM), Centre de recherche en épidémiologie et santé des populations (CESP), U1018, 94805, Villejuif, France

**Keywords:** Survie, Cancer primitif du foie, Facteurs pronostiques, Mortalité, Virus hépatite B, Virus hépatite C, Cotonou, Bénin, Afrique subsaharienne, Survival, Primary liver cancer, Prognostic factors, Mortality, hepatitis B Virus, Hepatitis C Virus, Cotonou, Benin, Sub-Saharan Africa

## Abstract

**Introduction:**

Le cancer primitif du foie (CPF) occupait la 6e et la 3e place dans le monde respectivement en termes d'incidence et de mortalité en 2020. L'objectif de ce travail était d’étudier la survie et les facteurs pronostiques du cancer primitif du foie à Cotonou en République du Bénin.

**Matériels et méthodes:**

Il s'est agi d'une étude de cohorte rétrospective qui a inclus les enregistrements de 150 patients atteints d'un CPF, répertoriés par le Registre des cancers de Cotonou, sur une période de sept ans allant du 1er janvier 2014 au 31 décembre 2020. Le logiciel R 3.6.1 a été utilisé pour l'analyse des données. La méthode de Kaplan-Meier a permis d'estimer la survie des patients. La comparaison des courbes de survie a été faite par le test du Log-Rank. Le modèle de Cox à risque proportionnel a été établi pour identifier les facteurs prédictifs de la mortalité. Le seuil de significativité statistique a été fixé à 5 %.

**Résultats:**

L’âge moyen des sujets était de 51,7 ± 14,9 ans et le sex-ratio de 2,7. La moitié des décès était survenue dans les deux premiers mois ayant suivi le diagnostic. Les facteurs pronostiques, après l'analyse multivariée, étaient: l’âge ≥ 60 ans (HRa = 1,7; IC 95 % [1,10-2,51]), la notion de consommation d'alcool (Hazard Ratio ajusté, HRa = 3,7; [1,33-9,42]), l'itinéraire thérapeutique (HRa = 1,9; [1,24-3,02]), l'infection par le virus de l'hépatite B (HRa = 7,7; [3,26-12,29]), l'infection par le virus de l'hépatite C (HRa = 3,6; [1,38-9,43]) et le délai de consultation ≥4 semaines (HRa = 2,0; [1,01-4,05]).

**Conclusion:**

La mortalité des patients atteints de CPF à Cotonou est élevée avec une médiane de survie de deux mois. Des facteurs, pour l'essentiel modifiables, sont associés à cette mortalité.

## Abstract (en)

**Introduction:**

Primary liver cancer (PLC) occupied the 6th and 3rd place in the world respectively in terms of incidence and mortality in 2020. The objective of this work was to study the survival and prognostic factors of primary liver cancer from 2014 to 2020 in Cotonou.

**Materials and methods:**

This retrospective cohort study included 150 patients registered with primary liver cancer, in the database of the Cotonou Cancer Registry, over a seven-years period from 01/01/2014 to 12/31/2020. R 3. 6. 1 software was used for data analysis. The Kaplan-Meier method was used to estimate patient survival. The comparison of the survival curves was made by the Log-Rank test. The Cox proportional hazard model was established to identify predictors of mortality. The statistical significance threshold was set at 5%.

**Results:**

The mean age of the subjects was 51. 7 ± 14. 9 years and the sex-ratio 2. 7. Half of the deaths occurred within the first 2 months after diagnosis. The prognostic factors, after multivariate analysis, were: age ≥ 60 (Hazard Ratio adjusted, HRa = 1. 7; IC 95% [1. 10-2. 51]), alcohol consumption (HRa = 3. 7; [1. 33-9. 42]), therapeutic itinerary (HRa = 1. 9; [1. 24-3. 02]), hepatitis B virus infection (HRa = 7. 7; [3. 26-12. 29]), hepatitis C virus infection (HRa = 3. 6; [1. 38-9. 43]) and time to consultation ≥4 weeks (HRa = 2. 0; [1. 01-4. 05]).

**Conclusion:**

The mortality of patients with CPF in Cotonou is high with a median survival of 2 months. Factors, mostly modifiable, are associated with this mortality.

## Introduction

Selon les statistiques du Centre international de recherche sur le cancer (CIRC), au Bénin en 2020, le cancer primitif du foie (CPF) occupait la 5^e^ place en termes d'incidence avec une proportion de 7,4 %. Le CPF était en outre le 2^e^ cancer chez l'homme et le 5^e^ chez la femme, avec un *sex-ratio* de 2,1 [11]. Dans la même période, l'Afrique subsaharienne, avec plus de 38 000 nouveaux cas de CPF était la quatrième région la plus affectée au monde après l'Asie du Sud-Est, l'Asie centrale du Sud et l'Amérique du Nord [11]. En 2020, le CPF était également le premier cancer digestif en milieu hospitalier à Cotonou (38,3 %) [19].

Le poids de cette affection augmente avec une prédilection pour certains sous-groupes de la population [32]. Les taux de mortalité les plus élevés sont observés en Afrique [12, 13, 21, 25, 27, 29, 30, 37]. La variation globale de l'incidence du CPF entre régions peut notamment s'expliquer par la distribution de l'infection par les virus des hépatites B et C. Ces deux virus sont responsables à eux seuls de 78 % de la mortalité liée au cancer du foie dans le monde [4, 13, 15, 27, 37].

Du fait de son traitement extrêmement coûteux, le CPF entraîne des pertes de ressources économiques et d'opportunités pour les patients, les familles, les employeurs et la société en général. Ces pertes comprennent des pertes financières, une qualité de vie réduite et des décès prématurés [35]. Malgré la mise en œuvre de traitements potentiellement curatifs tels que la résection hépatique, l'ablation par radiofréquence et la transplantation hépatique, le pronostic est encore généralement mauvais même dans les pays occidentaux [6, 8, 26, 36]. Le taux de survie à cinq ans des personnes atteintes du CPF reste inférieur à 80 % quel que soit le type de traitement reçu [33] et dépend du stade atteint au moment du diagnostic [24]. Dans les pays en voie de développement comme le Bénin, le diagnostic du CPF est fait à des stades tardifs avec une survie nettement plus faible [20]. Au Bénin, nous disposons de peu de données sur les indicateurs (incidence, mortalité et survie) relatifs aux personnes atteintes du CPF, en dépit de la connaissance de la variation internationale substantielle de ces indicateurs [2, 3, 25, 31]. Ces données sont pourtant indispensables pour évaluer et améliorer les pratiques, évaluer les politiques, mobiliser les ressources en vue d'infléchir la tendance de la mortalité prématurée due au CPF. L'objectif de ce travail est d’étudier la survie et les facteurs pronostiques du CPF à Cotonou de 2014 à 2020.

## Matériel et méthodes

Le Registre des cancers de Cotonou est un registre populationnel des cancers qui couvre la ville de Cotonou, capitale économique du Bénin, dont la superficie est de 79 km^2^ pour une population estimée entre 680 000 et 692 000 habitants, entre 2014 et 2020. Le registre utilise le logiciel CanNReg5^1^ développé par le Centre international de recherche sur le cancer (CIRC) pour la saisie des données et pour les contrôles et vérifications. Il s'est agi d'une étude observationnelle analytique de type cohorte rétrospective. Cette étude a porté sur les enregistrements de patients diagnostiqués d'un CPF, et répertoriés par le Registre des cancers de Cotonou, entre le 1^er^ janvier 2014 et le 31 décembre 2020. La population d’étude était composée de tous les patients résidant à Cotonou dont le CPF a été diagnostiqué entre le 1^er^ janvier 2014 et le 31 décembre 2020. Étaient exclus tous les patients qui présentaient un autre cancer simultanément. Entre le 1^er^ janvier 2014 et le 31 décembre 2020,150 cas de CPF ont été diagnostiqués et documentés dans la base de données du Registre des cancers de Cotonou. Nous avons procédé à un échantillonnage exhaustif. Les enregistrements ont été retracés depuis le registre jusqu’à la source de l'enregistrement. Les informations ont été vérifiées ou mises à jour. Deux techniques de collecte de données ont été utilisées. Il s'agissait d'un dépouillement à l'aide d'une fiche d'enquête et d'entrevues structurées à l'aide d'un questionnaire standardisé. Du 1^er^ juin au 31 juillet 2021, les dossiers des patients étaient recherchés dans les sources (hôpitaux et cliniques). Pour les patients perdus de vue dans ces centres, leurs proches ont été contactés au téléphone lorsque le contact téléphonique était renseigné. Une visite à domicile était organisée si nécessaire. Toutes les données ont été vérifiées par un médecin spécialiste en cancérologie qui supervisait également les appels téléphoniques et les visites à domicile.

La variable d'intérêt était la « survie des patients atteints de CPF ». Les différentes variables indépendantes ont été regroupées par catégorie de facteurs comme suit: facteurs sociodémographiques (âge, sexe, milieu de résidence, niveau d'instruction, profession, religion, situation matrimoniale); facteurs étiologiques (consommation d'alcool, consommation de tabac, infections par le virus de l'hépatite B (VHB) ou le virus de l'hépatite C (VHC); facteurs liés au traitement (accès aux soins, recours à la médecine alternative et complémentaire, délai de consultation).

Les données ont été traitées et analysées à l'aide du logiciel R© version 3. 6. 1. Pour les variables quantitatives, des moyennes avec leur écart-type, lorsque la distribution était normale, ont été exprimées. Pour les variables qualitatives, les effectifs et leur pourcentage ont été présentés. La survie a été évaluée par la méthode de Kaplan-Meier. Le test non paramétrique du log-rank a été utilisé pour comparer statistiquement les courbes de survie. Pour l'analyse multivariée, le modèle semi-paramétrique de Cox à risque proportionnel a été utilisé pour identifier les facteurs associés à la survie. Le *Hazard Ratio* ajusté (HRa) assorti des intervalles de confiance à 95 % a été utilisé comme mesure d'association pour quantifier la force des associations. Le seuil de significativité statistique a été fixé à 5 %. Cette étude a utilisé des données anonymisées provenant de la base de données du Registre des cancers de Cotonou. Des autorisations ont été obtenues auprès du Programme national de lutte contre les maladies non transmissibles (PNLMNT) et du Registre des cancers de Cotonou. L'accès à la base de données produite a été limité aux seules personnes autorisées. Cette base de données était conservée dans un ordinateur, et protégée par un nom d﹥utilisateur et un mot de passe. Les fichiers ont été rendus anonymes et codés avant tout transfert par support électronique. Les noms des patients ont été remplacés par des codes. De même, les modalités des variables ont été codifiées.

## Résultats

Au total, du 1^er^ janvier 2014 au 31 décembre 2020,150 patients répondant aux critères d'inclusion ont été enregistrés dans la base de données du Registre des Cancers de Cotonou.

### Facteurs sociodémographiques

**Figure 1:**
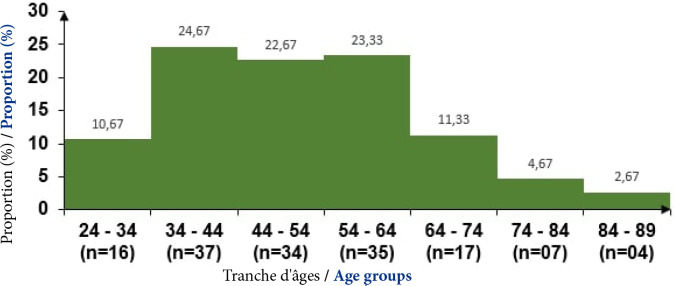
Répartition des 150 patients atteints de CPF selon les tranches d’âges (Cotonou, 2014-2020)

L’âge moyen des sujets était de 51,7±14,9 ans avec des extrêmes de 24 et 89 ans. La fréquence maximale était observée entre 34 et 44 ans (24,7 %; n = 37), (Fig. 1). Cent dix patients étaient de sexe masculin (73,3 %), soit un sex-ratio de 2,7. Le niveau d'instruction a pu être précisé chez 139 patients. Au total, 30,9 % des sujets n'avaient aucune instruction scolaire (n=43; 30,9 %). La profession avait été précisée chez 139 patients. Quatre-vingtdix-huit sujets (soit 70,5 %) travaillaient en indépendant ou dans le secteur informel. Le Tableau I présente les caractéristiques sociodémographiques des patients atteints du CPF à Cotonou de 2014 à 2020 selon la profession (n = 139).

**Tableau I:** Répartition des patients atteints de CPF selon le sexe, le niveau d'instruction, la situation matrimoniale (Cotonou, 2014-2020)

	Fréquence	
	absolue	relative (%)
**Sexe**	n = 150	
**homme**	110	73,3
**femme**	40	26,7
**Niveau d'instruction**	n = 139	
**aucun**	43	30,9
**primaire**	44	31,7
**secondaire**	21	15,1
**supérieur**	31	22,3
**Situation matrimonial**	n = 139	
**célibataire**	01	0,7
**marié**	137	98,6
**séparé**	01	0,7
**Profession**	n = 139	
**étudiant/élève**	02	1,44
**fonctionnaire**	11	7,91
**cadre du secteur privé**	**22**	15,8
**indépendant/secteur informel**	98	70,5
**retraité**	06	4,3

### Facteurs étiologiques

La consommation d'alcool et de tabac, l'infection par le VHB et l'infection par le VHC avaient été précisées chez 139 patients. Quatre sujets sur cinq, soit 81,3 % (n = 113) consommaient de l'alcool. Deux sujets sur 10 soit 20,9 % (n = 29) avaient consommé du tabac. Une infection par le virus de l'hépatite C était retrouvée chez 30,9 % (n=43). Plus de la moitié des sujets avait une infection par le virus de l'hépatite B (67,6 %; n = 94). (Tableau II). De plus, huit patients (5,3 %) avaient une co-infection VHB-VHC. La co-infection avec le virus de l'immunodéficience humaine et l'exposition à l'aflatoxine B1 n'ont pas pu être précisées.

**Tableau II:** Répartition des patients atteints de CPF selon les facteurs étiologiques (Cotonou, 2014-2020)

	Fréquence	
	absolue	relative (%)
**Notion de consommation d'alcool**		
**oui**	113	81,3
**non**	26	18,7
**Infection par le VHB**		
**oui**	94	67,6
**non**	45	32,4
**Infection par le VHC**		
**oui**	43	30,9
**non**	96	69,1
**Notion de consommation de tabac**		
**oui**	29	20,9
**non**	110	79,1

### Facteurs liés au traitement

L'information sur le recours à la médecine alternative et complémentaire avait été obtenue chez 139 patients. Ainsi, 49,6 % (n = 69) des patients ont eu recours à cette forme de médecine avant l'admission à l'hôpital. Chez 139 patients, les informations sur le délai séparant l'apparition des premiers symptômes et la date de la première consultation avaient été obtenues. En moyenne, ce délai était de 4,5±2,0 semaines avec les extrêmes d'une et sept semaines. Des 139 patients chez qui l'information était disponible, deux sujets, soit 1,4 % ont eu accès à des soins à visée curative *versus* 98,6 % (n = 137) qui avaient reçu des soins à visée palliative. Les deux patients traités à visée curative avaient bénéficié d'une résection hépatique.

### Survie des patients

**Figure 2:**
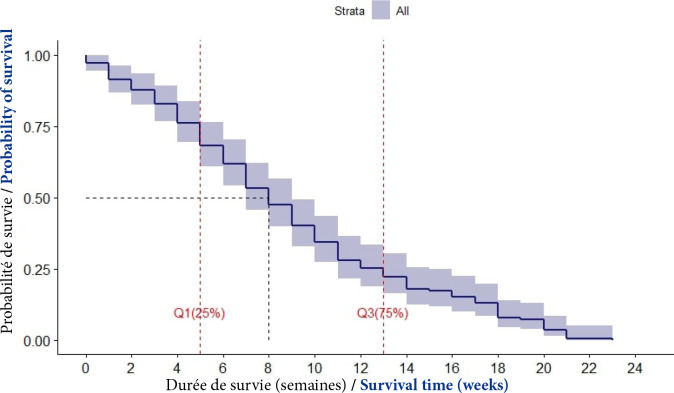
Courbe de survie globale des patients atteints de CPF (Cotonou, 2014-2020)

La médiane de survie après le diagnostic était de huit semaines avec un intervalle interquartile (IIQ) de [5, 6, 7, 8, 9, 10, 11, 12, 13] semaines (Fig. 2).

### Analyse univariée

#### Facteurs sociodémographiques associés à la survie

##### Sexe

**Figure 3:**
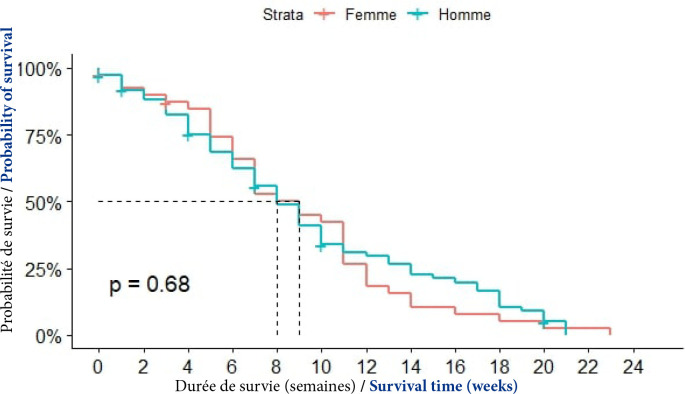
Courbes de survie des patients atteints de CPF selon le sexe à Cotonou de 2014 à 2020 (n = 150)

La médiane de survie était de 8 semaines chez les hommes et de 9 semaines chez les femmes. La différence n’était pas statistiquement significative (p = 0,68), (Fig. 3).

##### Age

**Figure 4:**
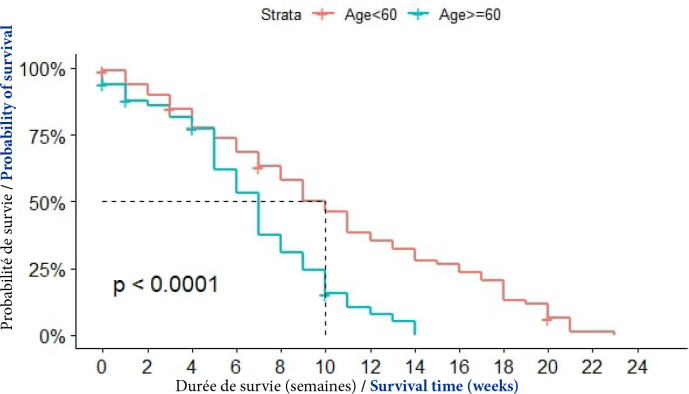
Courbes de survie des patients atteints de CPF selon la tranche d’âge à Cotonou de 2014 à 2020 (n=150)

La médiane de survie était de 10 semaines chez les patients ayant moins de 60 ans. Elle était de 7 semaines chez ceux qui avaient plus de 60 ans. La différence était statistiquement significative (p < 0,0001), (Fig. 4).

##### Niveau d'instruction

**Figure 5:**
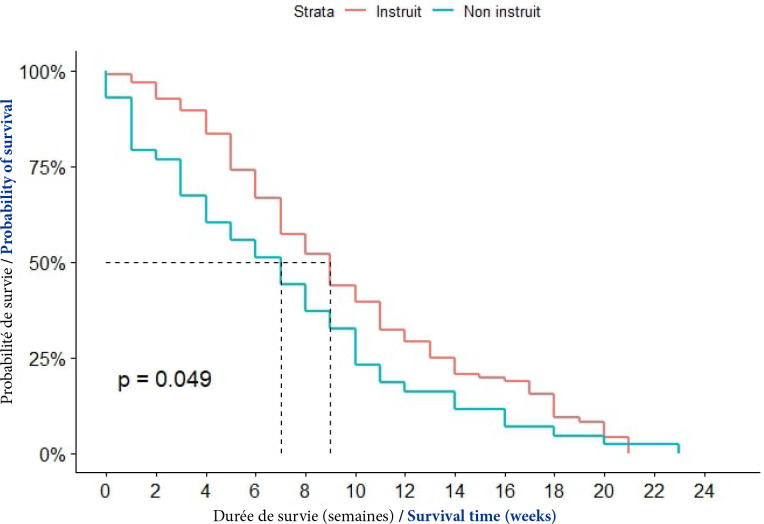
Courbes de survie des patients atteints de CPF selon le niveau d’éducation scolaire à Cotonou de 2014 à 2020 (n = 139)

Chez les patients qui n'avaient aucune éducation scolaire, la médiane de survie était de 7 semaines; elle était de 9 semaines chez les patients ayant une éducation scolaire. La probabilité de survie était significativement différente selon le niveau d’éducation scolaire (p = 0,049), (Fig. 5).

#### Facteurs étiologiques associés à la survie

##### Notion de consommation d'alcool

**Figure 6:**
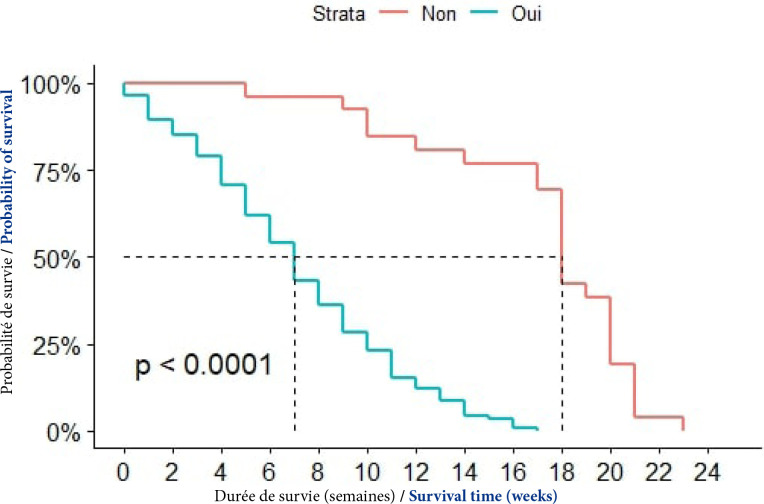
Courbes de survie des patients atteints de CPF selon la notion de consommation d'alcool à Cotonou de 2014 à 2020 (n = 139)

Chez les patients qui avaient une notion de consommation d'alcool, la médiane de survie était de 7 semaines contre 18 semaines chez les autres. La prise d'alcool influençait significativement la survie des patients (p< 0,0001), (Fig. 6).

##### Notion de consommation de tabac

**Figure 7:**
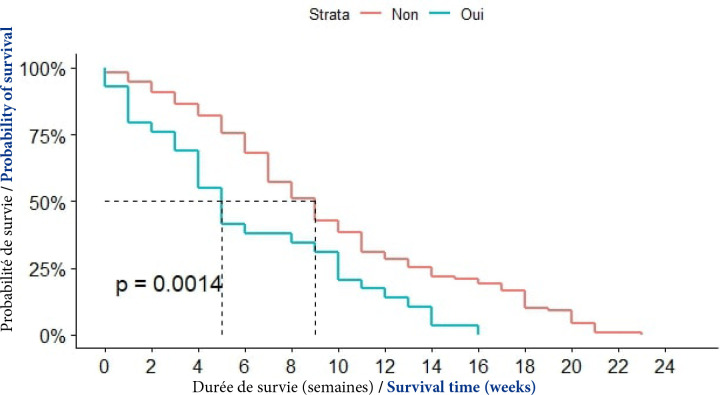
Courbes de survie des patients atteints de CPF selon la notion de consommation de tabac à Cotonou de 2014 à 2020 (n = 139)

La médiane de survie était de 5 semaines après le diagnostic chez les patients tabagiques; elle était de 9 semaines chez les patients ne consommant pas de tabac. Cette différence était statistiquement significative (p = 0,0014), (Fig. 7).

##### VHC

**Figure 8:**
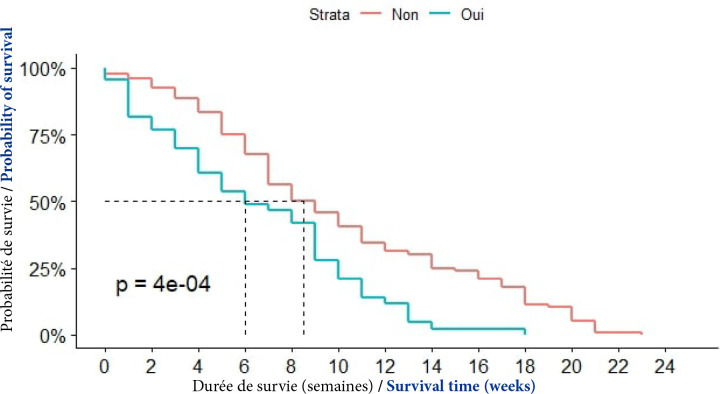
Courbes de survie des patients atteints de CPF en fonction de l'infection par le VHC à Cotonou de 2014 à 2020 (n = 139)

En présence de l'infection par le VHC, 50 % des patients étaient décédés au bout de 6 semaines après le diagnostic. Cette proportion était observée, en l'absence de l'infection par le VHC, au bout de 9 semaines, (p=0,0004) (Fig. 8).

##### VHB

**Figure 9:**
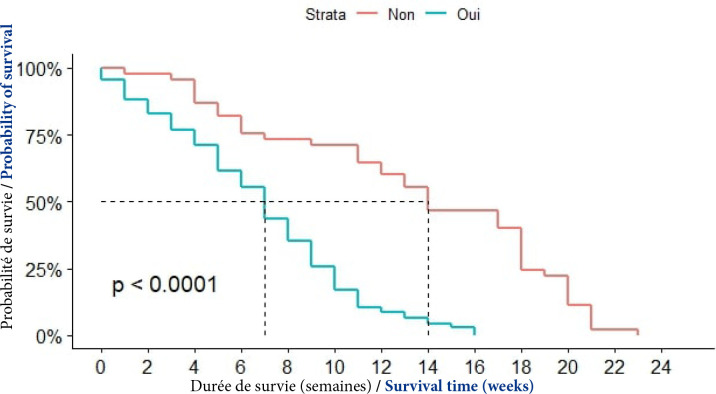
Courbes de survie des patients atteints de CPF selon l'infection par le VHB à Cotonou de 2014 à 2020 (n = 139)

Dans le groupe des patients infectés par le VHB, la médiane de survie était de 7 semaines; elle était de 14 semaines chez les sujets non infectés par le VHB. La différence était statistiquement significative (p<0,0001), (Fig. 9).

#### Facteurs thérapeutiques associés à la survie

##### Accès aux soins

La moitié (50 %) des patients était décédée au bout de 8 semaines après le diagnostic parmi ceux qui avaient reçu des soins à visée palliative, vs 22 semaines pour ceux qui avaient eu des soins à visée curative. La différence était statistiquement significative (p<0,0001).

##### Recours à la médecine alternative et complémentaire

**Figure 10:**
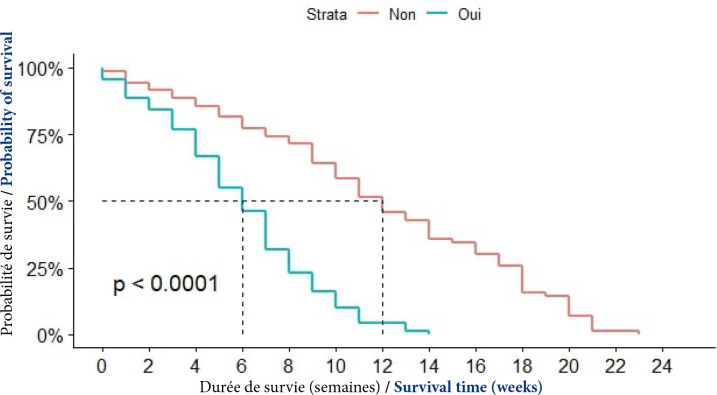
Courbes de survie des patients atteints de CPF selon le recours à la médecine alternative et complémentaire à Cotonou de 2014 à 2020 (n = 139)

En cas de recours initial à la médecine alternative, la médiane de survie était de 6 semaines. Elle était de 12 semaines chez ceux qui n'avaient pas eu recours à cette forme de médecine. La différence était statistiquement significative (p<0,0001), (Fig. 10).

##### Délai de consultation

**Figure 11:**
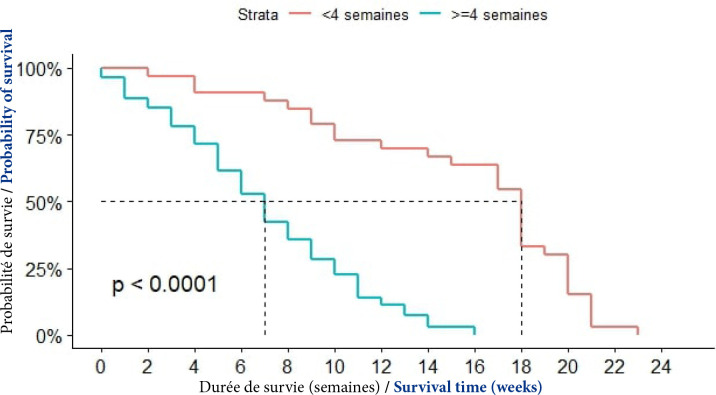
Courbes de survie des patients atteints du CPF selon le délai de consultation à Cotonou de 2014 à 2020 (n = 139)

La moitié (50 %) des décès était survenue au bout de sept semaines chez les patients ayant fait leur première consultation après quatre semaines suivant l'apparition des premiers symptômes. Dans les autres cas (moins de quatre semaines), cette proportion était observée au bout de 18 semaines. La différence était statistiquement significative (p<0,0001), (Fig. 11).

### Analyse multivariée

Après élimination progressive des variables dont le degré de signification était supérieur ou égal à 5 %, l’âge, la consommation d'alcool, le recours à la médecine alternative et complémentaire, l'infection par le VHB, l'infection par le VHC et le délai de consultation ont été retenus comme facteurs associés significativement à la survie chez les patients atteints de CPF dans le modèle final (Tableau III). Les patients atteints de CPF et âgés de plus de 60 ans couraient 1,7 fois plus de risque de décéder que ceux de moins de 60 ans. Les patients qui consommaient de l'alcool couraient 3,7 fois plus de risque de décéder que ceux qui n'en consommaient pas. Le risque de décéder dans le groupe des patients ayant eu recours à la médecine alternative ou complémentaire était 1,9 fois supérieur à celui des patients qui n'y avaient pas eu recours. Le risque de décéder était 7,7 fois plus élevé chez les patients infectés par le VHB que chez ceux qui ne l’étaient pas. De même, chez les patients infectés par le VHC, le risque de décéder était 3,6 fois plus élevé que parmi les patients non infectés par le VHC. Parmi les patients ayant eu leur première consultation hospitalière plus de quatre semaines après le début des symptômes, le risque de décéder était deux fois plus élevé que celui des autres patients.

**Tableau III:** Facteurs associés à la survie des patients atteints de CPF selon le modèle semi-paramétrique de Cox (Cotonou, 2014- 2020)

	Survie		
	HRa	IC95 %	p-value
Sexe (homme/femme*)	1,0	0,68-1,58	0,8569
Age (≥60/<60*)	1,7	1,10-2,51	0,0166**
Niveau d'instruction (non scolarisés/scolarisés*)	1,0	0,67-1,57	0,9202
Notion de consommation d'alcool	3,7	1,33-9,42	0,0126**
Notion de consommation de tabac (oui/non*)	1,7	0,39-2,28	0,1664
Accès aux soins (soins à visée curative /soins à visée palliative*)	0,2	0,02-1,20	0,0743
Recours à la médecine alternative et complémentaire (oui/non*)	1,9	1,24-3,02	0,0034**
Infection par le VHB (oui/non*)	7,7	3,26-12,29	0,0000**
Infection par le VHC (oui/non*)	3,6	1,38-9,43	0,0089**
Délai de consultation en semaines (≥4/<4*)	2,0	1,01-4,05	0,0458**

HR=Hazard Ratio

* Groupe de référence

** Significatif à l'analyse multivariée (p<5 %)

## Discussion

### Survie des patients atteints du CPF

Au terme de l’étude, la médiane de survie des patients atteints d'un CPF à Cotonou a été déterminée. Ainsi, de 2014 à 2020,50 % des patients de l’échantillon étaient décédés dans les huit premières semaines suivant la date du diagnostic. Cependant, le caractère rétrospectif de cette étude pourrait être à l'origine de biais. Cette étude, réalisée en milieu hospitalier dans la capitale du pays, pourrait ne pas être le reflet de la situation dans les autres régions du pays notamment les zones rurales où les patients ont un accès plus limité aux services de santé.

Une durée de survie médiane globale de 31 jours a été rapportée dans le nord-est du Nigéria en 2019 par Adamu *et al.* [1]. La survie médiane des patients atteints de CPF est de 10,9 mois en Égypte [36]. En Afrique du Sud, elle est de 6 semaines à compter du diagnostic et de 11,2 semaines à compter de l'apparition des symptômes [16]. En Gambie, la survie médiane des patients atteints de CPF est estimée à 91 jours [22]. Ces médianes de survie relativement faibles en Afrique au sud du Sahara sont similaires à celle qui a été déterminée dans cette étude (2 mois).

Cette survie est nettement meilleure en Chine, estimée à 25,2 mois dans une étude réalisée entre 2002 et 2012 par Xu *et al.* et publiée en 2015 [34]. Il faut noter que dans leur étude, sur un échantillon de 451 patients, 172 avaient subi une résection hépatique, 191 une chimio-embolisation artérielle hépatique et 88 une ablation par radiofréquence. En somme, 58 % (260/451) des patients inclus dans cette étude avaient bénéficié d'un traitement à visée curative. Dans la nôtre, deux patients avaient bénéficié de ce type de traitement. Cette meilleure survie retrouvée en Chine pourrait, au moins en partie, être attribuable à un meilleur accès au traitement à visée curative. Une survie médiane d'un an était rapportée dans une étude publiée en 2010 par Mathur *et al.* aux USA [28]. Au stade I de la maladie, la médiane de survie selon l'origine ethnique était de 18 mois chez les Asiatiques, 14 mois chez les Blancs, 12 mois chez les Hispaniques et 9 mois chez les Noirs. Au stade II de la maladie, cette médiane de survie était de 8 mois chez les Asiatiques, 6 mois chez les Blancs, 6 mois chez les Hispaniques et 5 mois chez les Noirs. La survie chez le sujet noir atteint de CPF semble relativement faible, même dans les pays occidentaux où existent des modalités thérapeutiques à visée curative.

Les médianes de survie des patients atteints d'un CPF en Afrique au sud du Sahara sont faibles parce que la maladie est généralement diagnostiquée au stade avancé et que les ressources thérapeutiques sont limitées. Dans un tel contexte, il est important que les politiques soient centrées sur la prévention, notamment par la vaccination contre le virus de l'hépatite B. La capacité de prise en charge des formations sanitaires doit être renforcée notamment pour : (i) l'identification des facteurs de risques d'hépatopathies; (ii) l’évaluation non invasive de la fibrose hépatique; (iii) le dépistage semestriel échographique du cancer primitif du foie chez les patients à risque; (iv) le développement de la chirurgie hépatique.

### Facteurs associés à la survie

Cette étude a également permis de déterminer les facteurs associés à la survie des patients atteints d'un CPF à Cotonou. Ainsi, les principaux facteurs pronostiques, après ajustement, étaient : l’âge (≥60 ans), la consommation d'alcool, le recours à la médecine alternative et complémentaire, l'infection par le VHB, l'infection par le VHC, le délai de consultation (≥4 semaines). Cependant, une des limites importantes de cette étude était liée à son caractère rétrospectif qui n'a pas permis d’évaluer avec précision tous les potentiels facteurs associés (par exemple la quantification précise de la consommation d'alcool et de tabac, le bilan biologique hépatique, le statut sérologique VIH, etc.). Ainsi, il convient de rappeler que plusieurs caractéristiques étiologiques (biologiques et cliniques) importantes pour la compréhension de la survie des patients n’étaient disponibles ni dans le registre des cancers, ni dans les observations médicales et de ce fait n'ont pu être recueillies dans le cadre de cette étude.

L'un des facteurs pronostiques le plus souvent retrouvé dans les études africaines est le stade atteint au moment du diagnostic. Selon Adamu *et al.,* la plupart des patients ne se font pas dépister à temps, car ils consultent pour la plupart des pharmaciens de bord de route, de village, des religieux et des herboristes [1]. Cette tendance a été confirmée dans notre étude où l'usage des thérapies alternatives et complémentaires était significativement associé à une augmentation de la mortalité. Ding *et al.* avaient trouvé, en 2019 en Chine, que le risque de décès par cancer du foie était multiplié par 1,4 lorsque l’âge augmentait de 5 ans [9]. Aux USA, il était multiplié par 1,8 lorsque l’âge dépassait 70 ans d'après Khalaf *et al.* en 2017 [17].

L'augmentation de l’âge est significativement associée à la baisse de la survie dans plusieurs études [1, 7, 9, 23]. Dans notre étude, la notion de consommation d'alcool était significativement associée au décès des patients. Lorsqu'il était consommé, les patients risquaient 3,7 fois plus le décès que les autres. Notre constat est confirmé par ceux d'autres auteurs. Ainsi, Ding *et al.* ont observé en Chine, en 2019, que la prise d'alcool augmentait le risque de décès de 17 % [9]. Brar *et al.* ont trouvé en 2020 aux USA que l'alcool augmentait de 33 % le risque de décès des patients atteints du CPF [5]. Dans plusieurs études, l'alcool est rapporté comme un facteur de risque d'une survenue rapide de décès chez les patients atteints du CPF [18, 23]. Significativement associé au décès des patients, le recours à la médecine alternative et complémentaire multiplie par 1,9 le risque de la survenue de cet événement. Dans notre étude, le risque de décès des patients atteints de CPF est significativement augmenté de 87 % en présence du VHB. C'est l'un des principaux facteurs de survenue du CPF [18, 9]. Tout comme l'infection par le VHB, le risque de décès des patients atteints de CPF est significativement augmenté de 72 % en présence de l'infection par le VHC. C'est également l'un des principaux facteurs de risque de survenue du CPF évoqué dans la plupart des études de survie aux côtés du VHB et de l'alcool [17, 23, 34]. Au Bénin, le vaccin contre l'hépatite B a été introduit, combiné en 2002 avec les valences DTC (Diphtérie, tétanos, coqueluche) et l'administration d'une dose à la naissance en 2020. Les patients de cette étude, âgés en moyenne de 51 ans, font probablement partie des générations qui n'ont pas été systématiquement vaccinées. Le risque de décès des patients atteints de CPF était, significativement, deux fois plus élevé chez les patients ayant eu une consultation hospitalière à plus de quatre semaines après l'apparition des premiers symptômes. Le sexe, le niveau d'instruction, la consommation de tabac et l'accès aux soins n’étaient pas associés aux décès des patients après l'analyse multivariée. Nous attribuons ce constat à la taille de notre échantillon qui est très inférieure à celles d'autres auteurs [5, 7, 9, 10, 14, 34, 38]. Ainsi, plusieurs facteurs influencent le pronostic des CPF dans notre contexte mais également dans plusieurs études à travers le monde. L'amélioration de la survie des patients ne peut se concevoir sans la mise en œuvre de politiques prenant en compte spécifiquement ces facteurs.

## Conclusion

Ce travail avait pour objectif d’étudier la survie et les facteurs pronostiques des patients atteints de CPF. Il en est ressorti que la probabilité de survivre après deux mois était de 50 %. Les constats faits dans cette étude montrent qu'il est indispensable de repenser les politiques sanitaires pour lutter plus efficacement contre ce problème de santé publique en Afrique au sud du Sahara. Il est urgent de renforcer notamment les actions tant au niveau de la prévention primaire (sensibilisation et éducation sur les facteurs de risques), que de la prévention secondaire (dépistage des hépatites pour un traitement précoce) pour réduire les impacts humains et socioéconomiques attribuables aux décès. Il est de plus indispensable de renforcer les politiques de prévention (poursuite du vaccin anti-VHB à la naissance et extension aux groupes à risque non immunisés notamment), d'améliorer le dépistage des hépatopathies et l'accès aux traitements pour réduire le risque de CPF et sa mortalité élevée. Par ailleurs, ces résultats appellent à de nouvelles études spécifiques sur l'accès aux soins des patients et sur l’évaluation de l'impact des modèles thérapeutiques sur la survie, afin de disposer de davantage d’éléments complémentaires d'actions pour infléchir la tendance de la mortalité précoce des patients atteints de CPF.

## Contribution des auteurs

Dismand Stephan Houinato, Freddy Houéhanou Rodrigue Gnangnon, Koffi N'Tcha, Aboudou Raïmi Kpossou ont conçu l'idée de l’étude. Freddy Houéhanou Rodrigue Gnangnon, Koffi N'Tcha Aboudou Raïmi Kpossou ont collecté les données. Koffi N'Tcha, Vincent Zossou, Cosme Toume, Salmane Amidou ont analysé les données. Dansou Gaspard Gbessi, Salmane Amidou, Jean Sehonou, Rodrigue S. Allodji ont contribué à l'interprétation et Freddy Houéhanou Rodrigue Gnangnon, Koffi N'Tcha et Aboudou Raïmi Kpossou ont rédigé l'article.

Tous les auteurs ont revu le manuscrit.

## Conflits d'intérêts

Les auteurs n'ont déclaré aucun conflit d'intérêt en rapport avec la présente étude.
