# Supplementary material for: Survie et facteurs pronostiques du cancer primitif du foie à Cotonou (République du Bénin) de 2014 à 2020
Source: Med Trop Sante Int. 2024 Jul 18;4(3):mtsi.v4i3.2024.418. [Article in French] doi: 10.48327/mtsi.v4i3.2024.418 (PMC11577100; doi:10.48327/mtsi.v4i3.2024.418)
Supplement: Related texts

# Research article: Survival and prognostic factors of primary liver cancer in Cotonou (Benin Republic) from 2014 to 2020

## Introduction

According to statistics from the International Agency for Research on Cancer (IARC), primary liver cancer (PLC) ranked 5th in terms of incidence in Benin in 2020, with a proportion of 7. 4%. It was also the 2nd most common cancer in men and 5th in women, with a sex ratio of 2. 1 [11]. During the same period, sub-Saharan Africa was the fourth most affected region in the world with more than 38,000 new cases of PLC, after Southeast Asia, South-Central Asia, and North America [11]. In 2020, PLC was also the leading digestive cancer in Cotonou hospitals (38. 3%) [19]. The burden of this disease is increasing, with a predilection for certain subpopulations [32]. The highest mortality rates are observed in Africa [12, 13, 21, 25, 27, 29, 30, 37]. The overall variation in the incidence of PLC between regions can be explained in particular by the distribution of infection with hepatitis B and C viruses. These two viruses alone are responsible for 78% of liver cancer mortality worldwide [4, 13, 15, 27, 37].

Due to its extremely costly treatment, PLC results in a loss of economic resources and opportunities for patients, families, employers and society in general. This includes financial losses, reduced quality of life and premature death [35].

Despite the introduction of potentially curative treatments such as liver resection, radiofrequency ablation and liver transplantation, the prognosis remains generally poor, even in Western countries [6, 8, 26, 36]. The five-year survival rate for people with PLC remains below 80% regardless of the type of treatment received [33] and depends on the stage at diagnosis [24]. In developing countries such as Benin, PLC is diagnosed at a late stage and survival is much lower [20].

In Benin, we have very little data on indicators (incidence, mortality, and survival) related to patients with PLC, despite the substantial international variation in these indicators that is well documented [2, 3, 25, 31]. However, these data are essential for the assessment and improvement of practices, the evaluation of policies, and the mobilization of resources with the objective of reversing the trend of premature mortality due to PLC. The objective of this study is to examine survival and prognostic factors for PLC in Cotonou between 2014 and 2020.

## Materials and methods

The Cotonou Cancer Registry is a population-based cancer registry covering the city of Cotonou, the economic capital of Benin, with an area of 79 km2 and an estimated population of between 680,000 and 692,000 between 2014 and 2020. The registry uses the CanReg5^1^ software developed by the International Agency for Research on Cancer (IARC) for data entry, checks, and verifications. This was a retrospective, cohort, observational, analytical study. The study focused on records of patients diagnosed with PLC and identified by the Cotonou Cancer Registry between January 1st, 2014 and December 31,2020. The study population consisted of all patients residing in Cotonou and diagnosed with PLC between January 1st, 2014 and December 31,2020. All patients with another cancer at the same time were excluded. Between January 1st, 2014 and December 31,2020,150 cases of PLC were diagnosed and documented in the Cotonou Cancer Registry database. We performed an exhaustive sampling. Records were traced from the registry to the source of the record. Information was verified or updated. Two data collection techniques were used. These were tabulation using a survey form and structured interviews using a standardized questionnaire. From June 1 to July 31,2021, patient records were searched in the sources (hospitals and clinics). For patients lost to follow-up in these centers, their relatives were contacted by telephone if telephone contact was available. If necessary, a home visit was organized. All data were reviewed by a cancer specialist who also supervised the telephone calls and home visits. The variable of interest was “survival of PLC patients”. The various independent variables were grouped by category of factor as follows: sociodemographic factors (age, sex, place of residence, level of education, occupation, religion, marital status); etiological factors (alcohol consumption, tobacco consumption, infection with hepatitis B virus (HBV) or hepatitis C virus (HCV)); treatment-related factors (access to care, use of alternative and complementary medicine, consultation timeframe).

Data were processed and analyzed using R software© version 3. 6. 1. For quantitative variables, means with standard deviations were expressed when the distribution was normal. For qualitative variables, numbers and percentages were presented. Survival was evaluated using the Kaplan-Meier method.

The nonparametric log-rank test was used to statistically compare survival curves. For multivariate analysis, the semi-parametric Cox proportional hazards model was used to identify factors associated with survival. The adjusted hazard ratio (HRa) with 95% confidence intervals was used as the measure of association to quantify the strength of the associations. The threshold for statistical significance was set at 5%.

This study used anonymized data from the Cotonou Cancer Registry database. Permission was obtained from the Programme national de lutte contre les maladies non transmissibles (PNLMNT) and the Cotonou Cancer Registry. Access to the created database was restricted to authorized persons only. The database was stored on a computer and protected by a username and password. Files were anonymized and encrypted before electronic transmission. Patient names were replaced with codes. Similarly, the terms of the variables were coded.

## Results

In total, from January 1,2014 to December 31,2020,150 patients meeting the inclusion criteria were registered in the Cotonou Cancer Registry database.

### Socio-demographic factors

The mean age of the subjects was 51.7±14.9 years, with extremes of 24 and 89 years. The highest frequency was observed between the ages of 34 and 44 years (24.7%; n=37) (Fig. 1). One hundred and ten patients were male (73.3%), resulting in a sex ratio of 2.7. Education level was available for 139 patients. A total of 30.9% had no formal education (n=43; 30.9%). Occupation was available for 139 patients. Ninety-eight patients (70.5%) were self-employed or worked in the informal sector. Table I shows the sociodemographic characteristics of PLC patients in Cotonou from 2014 to 2020 according to occupation (n=139).

**Figure 1:** Distribution of the 150 patients with primary liver cancer according to age groups (Cotonou, 2014-2020)

**Table I:** Distribution of primary liver cancer patients by gender, education level, marital status (Cotonou, 2014-2020)

	Frequency	
	absolute	relative
**Sex**	n = 150	
**male**	110	73,3
**female**	40	26,7
**Level of education**	n = 139	
**none**	43	30,9
**primary**	44	31,7
**secondary**	21	15,1
**higher**	31	22,3
**Marital status**	n = 139	
**single**	01	0,7
**married**	137	98,6
**separated**	01	0,7
**Profession**	n = 139	
**student/pupil**	02	1,44
**civil servant**	11	7,91
**private-sector executive**	**22**	15,8
**self-employed/informal sector**	98	70,5
**retired**	06	4,3

### Etiological factors

Alcohol and tobacco use, HBV infection, and HCV infection were reported for 139 patients. Four out of five subjects (81.3%) (n=113) consumed alcohol. Two out of 10 subjects or 20.9% (n=29) had used tobacco. Hepatitis C infection was found in 30.9% (n=43). More than half of the subjects were infected with hepatitis B virus (67.6%; n=94). (Table II). In addition, eight patients (5.3%) were co-infected with HBV-HCV. Co-infection with human immunodeficiency virus and exposure to aflatoxin B1 could not be specified.

**Table II:** Distribution of primary liver cancer patients according to etiological factors (Cotonou, 2014-2020)

	Frequency	
	absolute	relative
**Notion of alcohol consumption**		
**yes**	113	81,3
**no**	26	18,7
**HBV infection**		
**yes**	94	67,6
**no**	45	32,4
**HBC infection**		
**yes**	43	30,9
**no**	96	69,1
**Notion of tobacco consumption**		
**yes**	29	20,9
**no**	110	79,1

### Treatment-related factors

Information on the use of alternative and complementary medicine was obtained from 139 patients. Thus, 49.6% (n=69) of the patients had used this form of medicine prior to hospitalization. Information on the time between the onset of the first symptoms and the date of the first consultation was obtained for 139 patients. The mean time was 4.5±2.0 weeks, with extremes of one and seven weeks. Of the 139 patients for whom information was available, two subjects (1.4%) had access to curative care, whereas 98.6% (n=137) had access to palliative care. The two patients who received curative treatment had undergone liver resection.

### Patient survival

Median survival after diagnosis was eight weeks, with an interquartile range (IQR) of [5, 6, 7, 8, 9, 10, 11, 12, 13] weeks (Fig. 2).

**Figure 2:** Overall survival curve of patients with primary liver cancer (Cotonou, 2014-2020)

### Univariate analysis

#### Socio-demographic factors associated with survival

##### Gender

Median survival was eight weeks in men and nine weeks in women. The difference was not statistically significant (p=0.68), (Fig. 3).

**Figure 3:** Survival curves of PLC patients by sex in Cotonou from 2014 to 2020 (n = 150)

##### Age

Median survival was 10 weeks for patients under 60. It was 7 weeks in patients aged over 60. The difference was statistically significant (p<0.0001), (Fig. 4).

**Figure 4:** Survival curves of PLC patients according to age groups in Cotonou from 2014 to 2020 (n=150)

##### Education level

In patients with no formal education, median survival was 7 weeks; in patients with formal education, median survival was 9 weeks. The probability of survival was significantly different according to the level of school education (p=0.049), (Fig. 5).

**Figure 5:** Survival curves of PLC patients according to level of school education in Cotonou from 2014 to 2020 (n = 139)

#### Etiological factors associated with survival

##### Notion of alcohol consumption

In patients with any history of alcohol consumption, median survival was 7 weeks compared with 18 weeks in the others. Alcohol consumption significantly influenced patient survival (p<0.0001), (Fig. 6).

**Figure 6:** Survival curves of PLC patients according to alcohol consumption in Cotonou from 2014 to 2020 (n = 139)

##### Notion of tobacco consumption

The median survival was 5 weeks after diagnosis in patients who smoked, compared with 9 weeks in patients who did not smoke. This difference was statistically significant (p=0.0014), (Fig. 7).

**Figure 7:** Survival curves of PLC patients according to tobacco consumption in Cotonou from 2014 to 2020 (n = 139)

##### HCV

Infection with HCV was linked to a 50% mortality rate within 6 weeks of diagnosis. This proportion was observed after 9 weeks in the absence of HCV infection (p=0.0004), (Fig. 8).

**Figure 8:** Survival curves of PLC patients according to HCV infection in Cotonou from 2014 to 2020 (n = 139)

##### HBV

In the HBV-infected group, median survival was 7 weeks, compared with 14 weeks in the HBV-uninfected group. The difference was statistically significant (p<0.0001), (Fig. 9).

**Figure 9:** Survival curves of PLC patients according to HBV infection in Cotonou from 2014 to 2020 (n = 139)

#### Therapeutic factors associated with survival

##### Access to care

Half of patients who received palliative care died within 8 weeks of diagnosis, compared with 22 weeks for those who received curative care. The difference was statistically significant (p<0.0001).

##### Use of alternative and complementary medicine

When alternative medicine was first use, the median survival was 6 weeks. It was 12 weeks for those who had not used this form of medicine. The difference was statistically significant (p<0.0001), (Fig. 10).

**Figure 10:** Survival curves of PLC patients according to the use of alternative and complementary medicine in Cotonou from 2014 to 2020 (n = 139)

##### Consultation timeframe

Half (50%) of the deaths occurred after seven weeks in patients who first presented 4 weeks after the onset of symptoms. In the remaining cases (less than 4 weeks), this proportion was observed at 18 weeks. The difference was statistically significant (p<0.0001), (Fig. 11).

**Figure 11:** Survival curves of PLC patients according to consultation time in Cotonou from 2014 to 2020 (n = 139)

### Multivariate analysis

After successive elimination of variables with a significance level greater than or equal to 5%, age, alcohol consumption, use of alternative and complementary medicine, HBV infection, HCV infection and consultation timeframe remained as factors significantly associated with survival in patients with PLC in the final model (Table III).

**Table III:** Factors associated with survival of FPC patients according to the semi-parametric Cox model (Cotonou, 2014-2020)

	Survival		
	HRa	IC95 %	p-value
Gender (male/female*0)	1,0	0,68-1,58	0,8569
Age (≥60/<60*)	1,7	1,10-2,51	0,0166**
Education level (not in school/in school)	1,0	0,67-1,57	0,9202
Notion of alcohol consumption (yes/no*)	3,7	1,33-9,42	0,0126**
Notion of tobacco consumption (yes/no*)	1,7	0,39-2,28	0,1664
Access to care (curative care/palliative care*)	0,2	0,02-1,20	0,0743
Use of alternative and complementary medicine (yes/no*)	1,9	1,24-3,02	0,0034**
HBV infection (yes/no*)	7,7	3,26-12,29	0,0000**
HCV infection (yes/no*)	3,6	1,38-9,43	0,0089**
Consultation time in weeks (≥4/<4*)	2,0	1,01-4,05	0,0458**

HR=Hazard Ratio

* Reference group

** Significant in multivariate analysis (p<5%)

PLC patients over 60 years of age were 1.7 times more likely to die than those under 60 years of age. Patients who consumed alcohol were 3.7 times more likely to die than those who did not. The risk of death was 1.9 times higher in the group of patients who had used alternative or complementary medicine than in those who had not. The risk of death was 7.7 times higher in patients infected with HBV than in those who were not. Similarly, HCV-infected patients were 3.6 times more likely to die than HCV-uninfected patients. Patients who had their first hospital visit more than four weeks after the onset of symptoms were twice as likely to die as other patients.

## Discussion

### Survival of PLC patients

At the end of the study, the median survival of PLC patients in Cotonou was determined. From 2014 to 2020,50% of patients in the sample died within the first eight weeks of diagnosis. However, the retrospective nature of this study may be a source of bias. This study, conducted in a hospital in the capital city, may not reflect the situation in other regions of the country, particularly rural areas, where patients have more limited access to health services.

In 2019, Adamu *et al.* reported a median overall survival time of 31 days in north-east Nigeria [1]. In Egypt, median survival for PLC patients is 10.9 months [36].

In South Africa, it is 6 weeks from diagnosis and 11.2 weeks from symptom onset [16]. In the Gambia, median survival for PLC patients is estimated at 91 days [22]. These low survival rates in sub-Saharan Africa are similar to those found in this study (2 months).

This survival is significantly better in China, estimated at 25.2 months in a study conducted between 2002 and 2012 by Xu et al and published in 2015 [34]. It should be noted that in their study, out of a sample of 451 patients, 172 had undergone liver resection, 191 hepatic arterial chemoembolization and 88 radiofrequency ablation. Overall, 58% (260/451) of the patients included in this study had received curative treatment. In our study, two patients had received this type of treatment. This better survival in China may be due, at least in part, to better access to curative treatment. A study published in 2010 by Mathur *et al.* in the USA reported a median survival of one year [28]. For stage I disease, median survival by ethnicity was 18 months for Asians, 14 months for whites, 12 months for Hispanics, and 9 months for blacks. For stage II disease, median survival was 8 months for Asians, 6 months for whites, 6 months for Hispanics, and 5 months for blacks. Survival in black PLC patients appears to be relatively poor, even in Western countries where curative treatment modalities are available. Median survival rates for PLC patients in sub-Saharan Africa are low because the disease is generally diagnosed at an advanced stage and therapeutic resources are limited. In this context, it is important that policies focus on prevention, particularly through vaccination against the hepatitis B virus. The management capacity of health facilities needs to be strengthened, particularly with regard to (i) identification of risk factors for liver disease; (ii) non-invasive assessment of liver fibrosis; (iii) biannual ultrasound screening for primary liver cancer in patients at risk; and (iv) development of liver surgery.

### Factors associated with survival

This study also determined the factors associated with survival in PLC patients in Cotonou. After adjustment, the main prognostic factors were: age (≥60 years), alcohol consumption, use of alternative and complementary medicine, HBV infection, HCV infection, and consultation timeframe (≥4 weeks).

However, one of the major limitations of this study was its retrospective nature, which meant that it was not possible to accurately assess all the potential associated factors (e.g. precise quantification of alcohol and tobacco consumption, liver biology work-up, HIV serological status, etc.). It should therefore be remembered that a number of etiological (biological and clinical) characteristics that are important for understanding patient survival were not available either in the cancer registry or in the medical observations, and so could not be collected as part of this study.

One of the prognostic factors most often found in African studies is the stage reached at diagnosis. According to Adamu et al, most patients do not undergo screening in time, as they mostly consult roadside or village pharmacists, clerics and herbalists [1]. This trend was confirmed in our study, where the use of alternative and complementary therapies was significantly associated with an increase in mortality. In China in 2019, Ding *et al.* found that the risk of death from liver cancer increased by a factor of 1.4 when age increased by 5 years [9]. In the USA, it increased by a factor of 1.8 when age exceeded 70, according to Khalaf *et al.* in 2017 [17].

Increasing age has been significantly associated with reduced survival in several studies [1, 7, 9, 23]. In our study, alcohol consumption was significantly associated with patient death. When alcohol was consumed, patients were 3.7 times more likely to die than others. Our findings are confirmed by those of other authors. Ding *et al.* observed in China in 2019 that alcohol consumption increased the risk of death by 17% [9]. Brar *et al.* found in 2020 in the USA that alcohol increased the risk of death in PLC patients by 33% [5]. In several studies, alcohol has been reported as a risk factor for the rapid onset of death in PLC patients [18, 23]. The use of alternative and complementary medicine is significantly associated with an increased risk of patient death. In our study, the risk of death in PLC patients was significantly increased by 87% in the presence of HBV [18, 9]. This is one of the main factors in the development of PLC. Similarly, the risk of death in PLC patients is significantly increased by 72% in the presence of HCV infection. HCV is also one of the main risk factors for the development of PLC in most survival studies, alongside HBV and alcohol [17, 23, 34]. In Benin, the hepatitis B vaccine was introduced in 2002, administered in conjunction with DTP (diphtheria, tetanus, and pertussis) at birth. The patients in this study, with an average age of 51, are likely to have been born before the introduction of the vaccine. The risk of death in PLC patients was significantly higher in those who had a hospital consultation more than four weeks after the onset of the first symptoms. After multivariate analysis, sex, level of education, smoking habits, and access to care were not found to be associated with patient death. This finding may be attributed to the relatively small size of our sample compared to those of other authors [5, 7, 9, 10, 14, 34, 38].

Several factors influence the prognosis of PLC in our context, but also in several studies around the world. Improving patient survival is inconceivable without implementing policies that specifically take these factors into account.

## Conclusion

The aim of this study was to investigate survival and prognostic factors in PLC patients. It showed that the probability of survival after two months was 50%. The findings of this study show that it is essential to rethink health policies in order to combat this public health problem more effectively in sub-Saharan Africa. There is an urgent need to step up action on both primary prevention (raising awareness and educating people about risk factors) and secondary prevention (screening for hepatitis so that it can be treated early) to reduce the human and socio-economic impact of deaths. In addition, it is vital to strengthen prevention policies (in particular, the continuation of HBV vaccination at birth and its extension to non-immunized at-risk groups), and to improve screening for liver disease and access to treatment in order to reduce the risk of PLC and its high mortality rate. In addition, these results call for further specific studies on access to care for patients and assessment of the impact of therapeutic models on survival, in order to provide additional evidence for action to reverse the trend towards early mortality in PLC patients.

## Authors’ contributions

Dismand Stephan Houinato, Freddy Houehanou Rodrigue Gnangnon, Koffi N'Tcha, Aboudou Raimi Kpossou conceived the idea for the study. Freddy Houéhanou Rodrigue Gnangnon, Koffi N'Tcha Aboudou Raïmi Kpossou collected the data. Koffi N'Tcha, Vincent Zossou, Cosme Toume, Salmane Amidou analyzed the data. Dansou Gaspard Gbessi, Salmane Amidou, Jean Sehonou, Rodrigue S. Allodji contributed to the interpretation and Freddy Houéhanou Rodrigue Gnangnon, Koffi N'Tcha and Aboudou Raïmi Kpossou wrote the article.

All authors reviewed the manuscript.

## Conflicts of interest

The authors have reported no conflicts of interest related to this study.
